# 

**Published:** 1906-05-05

**Authors:** 


No. 1,023. Vol. XL. [Registered as a Newspaper.] Saturday, May 5th, 1906. [Subscription Terms see p. 96.] PRICE THREEPENCE

